# Voltammetric Assay of Mercury Ion in Fish Kidneys

**DOI:** 10.5487/TR.2008.24.1.023

**Published:** 2008-03-01

**Authors:** Suw Young Ly

**Affiliations:** Biosensor Research Institute, Seoul National University of Technology, Seoul, 139-743 Korea

**Keywords:** Hg(II), DNA, Carbon nanotube, Voltammetry, Fish kidney

## Abstract

Voltammetric analysis of mercury ions was developed using paste electrodes (PEs) with DNA and carbon nanotube mixed electrodes. The optimized analytical results of the cyclic voltammetry (CV) of the 1∼14 ng L^−1^ Hg(II) concentration and the square wave (SW) stripping voltammetry of the 1∼12 ng L^−1^ Hg(II) working range within an accumulation time of 400 seconds were obtained in 0.1 M NH_4_H_2_PO_4_ electrolyte solutions of pH 4.0. For the relative standard deviations of the 1 ng L^−1^ Hg(II), which were observed at 0.078% (n = 15) at the optimum conditions, the low detection limit (S/N) was pegged at 0.2 ng L^−1^ (7.37 × 10^−13^M) for Hg(II). The results can be applied to assays in biological fish kidneys and wastewater samples.

## INTRODUCTION

In the aquatic ecosystem, mercury ions come from art paint, coal, food, pharmaceuticals, and others (Sang *et al*., 2004; Eun *et al*., 2003; Seung *et al*., 2003), which create trace metals that cause food or environmental toxicology (Joanna and Michael, 2000; Elsie and Gail, 2000; Nathalie *et al*., 2005). Their assay is particularly important in biological analyses (Janos and Erzsebet, 2005) such as for paresthesia, ataxia, dysarthyria, hearing defects, visual disturbances, and other medical fields (Carrington *et al*., 2004; Ertas and Tezel, 2004). Thus, various common analytical methods of mercury ion assay have been developed, such as gas chromatography - mass spectrometry (Giuseppe *et al*., 2004; Petru and Freddy, 2004), HPLC with atomic fluorescence spectrometry (Li *et al*., 2003, 2004), zeeman atomic absorption spectrometry (Sholupov *et al*., 2004), atomic absorption spectrometry (Cizdziel and Shawn, 2004; Shigehiro *et al*., 2004; Claudia *et al*., 2005), and others (Van Staden and Taljaard, 2004). These methods achieve very low detection ranges and are composed with various processing systems that involve separation, sample injection, temperature control, and detection, which depend on spectroscopic or voltammetric detection systems. Of late, more compact, simple, and sensitive analytical techniques are being required. Stripping voltammetry is sensitive in trace analysis, which depends on working electrode systems and is commonly used with drop mercury and mercury film electrodes (Clinio and Giancarlo, 2001; Tsai *et al*., 2001; Sonia, 1998; Percio *et al*., 2003; Joseph *et al*., 2001), glassy carbon electrodes (Joseph *et al*., 2000; Suw *et al*., 2002, 2004), paste electrodes (Tesfaye *et al*., 1999; Jahan *et al*., 2001; Korbut *et al*., 2001), and other modified electrodes. Despite this, few studies have been conducted on mercury analysis and, and these studies have achieved low detection limits. For example, the gold disk electrode arrived at 5 ng $L^{-1}$ after 10 min of deposition time (Ricardo *et al*., 2000); the electrochemical quartz crystal microbalance methods arrived at the 1 ppb level (Niels *et al*., 1996); the boron-doped diamond film electrode method arrived at the 0.005~50 ppb working range (Manivannan *et al*., 2005); and the nafion-coated glassy carbon electrode method reached a 10 nM detection limit (Zuliang *et al*., 1999). Some of these methods are used, however, with long accumulation times and have arrived at poor detection limits. Thus, in this study, new and more sensitive methods are investigated using paste electrodes (PEs) with DNA and carbon nanotube mixed electrodes. A former researcher has researched on the DNA-coated carbon paste electrode for drug detection (Radi, 1999), and the DNA-modified electrode responded distinctly with other molecules and ions (Yuan *et al*., 1999; Manli *et al*., 2004). On the other hand, carbon nanotube properties are useful in electrocatalysis and sensor applications [35] (Bailure *et al.*, 2003) and high electrical conductivity (Manli *et al.*, 2004), and can perform electron transfer with biomolecules (Gang *et al.*, 2002). In this study, the DNA and nanotube properties were combined in mercury ion analysis, which achieved optimum conditions at lower detection limits, thus registering better performance than other common voltammetric methods.

## EXPERIMENTAL PROCEDURE

All the voltammetric measurements in this study were made using a CHI660A instrument electrochemical workstation (from CH Instruments, Inc., Cordova, TN, USA). A three-electrode system was used to monitor the voltammogram. The PE was used as the working electrode and was saturated with Ag/AgCl/KCl as the reference, and a platinum wire was used as the auxiliary electrode. The electrolyte solutions were used with double-distilled water (18 M ohm $cm^{-1}$), and the double-stranded calf thymus DNA (dsDNA) and the other reagents were obtained from Aldrich. The multi-walled carbon nanotubes (15~40 nm in diameter) for the CVD method were obtained from Nanotech in Korea. Following this, the DNA and graphite nanotube mixing weight ratios of 5 : 1, 4 : 1, 3 : 1, 2 : 1, 1 : 1, 1 : 2, 1 : 3, 1 : 4, and 1 : 5 g were examined. At 1 : 2, the maximum increased peak current of 0.2 V Hg(II) appeared in cyclic voltammetry. Thus, the 1 : 2 ratio was used for all the experimental mixing weights. The paste electrode was made by mixing 70% powder with 30% mineral oil. This mixture was homogenized in a mortar for 30 minutes. The mixed paste was inserted into a plastic syringe with a diameter of 3.0 mm, and a copper wire was connected to the electrical system. The three-electrode system was immersed in a 15 m; cell that contained electrolyte solutions of 0.1 M ammonium phosphate solution, whereas the other parameters were maintained at optimal conditions. All the experiments were performed at a room temperature of 24 ± 0.5°C, without removing oxygen, and it was found that all the experiments could be performed in an open circuit. Various acid and base electrolyte solutions (all 0.1 M solutions) were initially examined in search of a possible supporting electrolyte. Ammonium phosphate solution was found to have been the most suitable.

## EXPERIMENT RESULTS

***Cyclic voltammetry.*** First, in the cyclic voltammetry, various concentration effects and peak potentials were studied using wide potential ranges with -1.0~1.0 V switching potentials and a scan rate of 0.5 V/s. Only oxidation peak signals were obtained at these conditions; reduction signals did not appear. Thus, various working ranges were examined, and very low concentration ranges were obtained at ppt levels of 10, 20, 30, 40, 50, 60, 70, 80, 90, and 100 ng $L^{-1}$ Hg(II) (not shown here). At these ranges, the linear equation y = 0.3168x + 4.893 and an $R^2$ = 9879 precision were sensitively obtained, the peaks of which sharply responded. More sensitive working ranges were examined using a faraday cage via environmental noise exclusion. Fig. 1 shows the very low ranges of 1~14 ng $L^{-1}$ Hg(II) with PE in an electrolyte solution, the very smooth blank solutions, and the non-appearance of noise peak signals; and that from 1 to 14 ng $L^{-1}$, sharply and linearly increasing signals were obtained. At these conditions, the slope ratio of $\Delta x/\Delta y$ = 5.50, the precision of $R^2$ = 0.9947, and the maximum peak height of 76.86 × $10^{-7}$ A appeared, and the peak width narrowly responded. These results are usable in any environment and biological application. More sensitive working ranges were searched for using square wave stripping voltammetry.

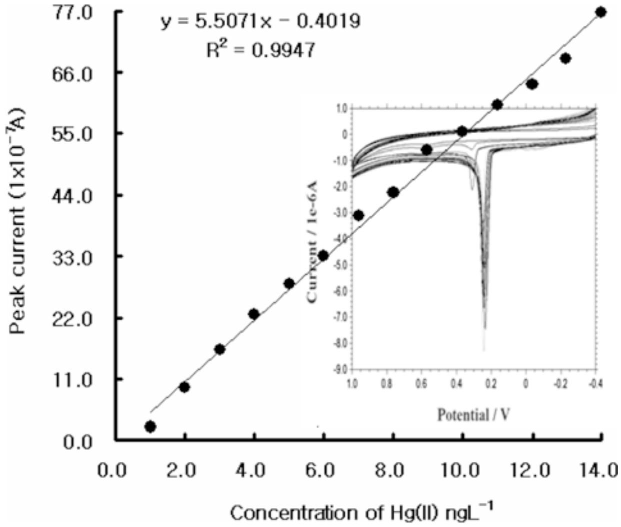

**Fig. 1.** Cyclic voltammetric concentration effects of 0, 1, 2, 3, 4, 5, 6, 7, 8, 9, 10, 11, 12, 13, and 14 ng $L^{-1}$ Hg(II) in pH 4.0 phosphate buffer solutions at a scan rate of 500 $mVs^{-1}$.

***Stripping voltammetry optimization.*** Fig. 2(a) shows the voltammetric peak current in the 100 mg $L^{-1}$ Hg(II) concentration as a function of varying square wave frequencies in the 50~500 Hz range, dswithin an accumulation time of 20 seconds. All the optimized examinations were used with short accumulation times for fast results at these conditions. Mercury ions were dispersed at the two peak potentials of 0.2 and 0.4 V,

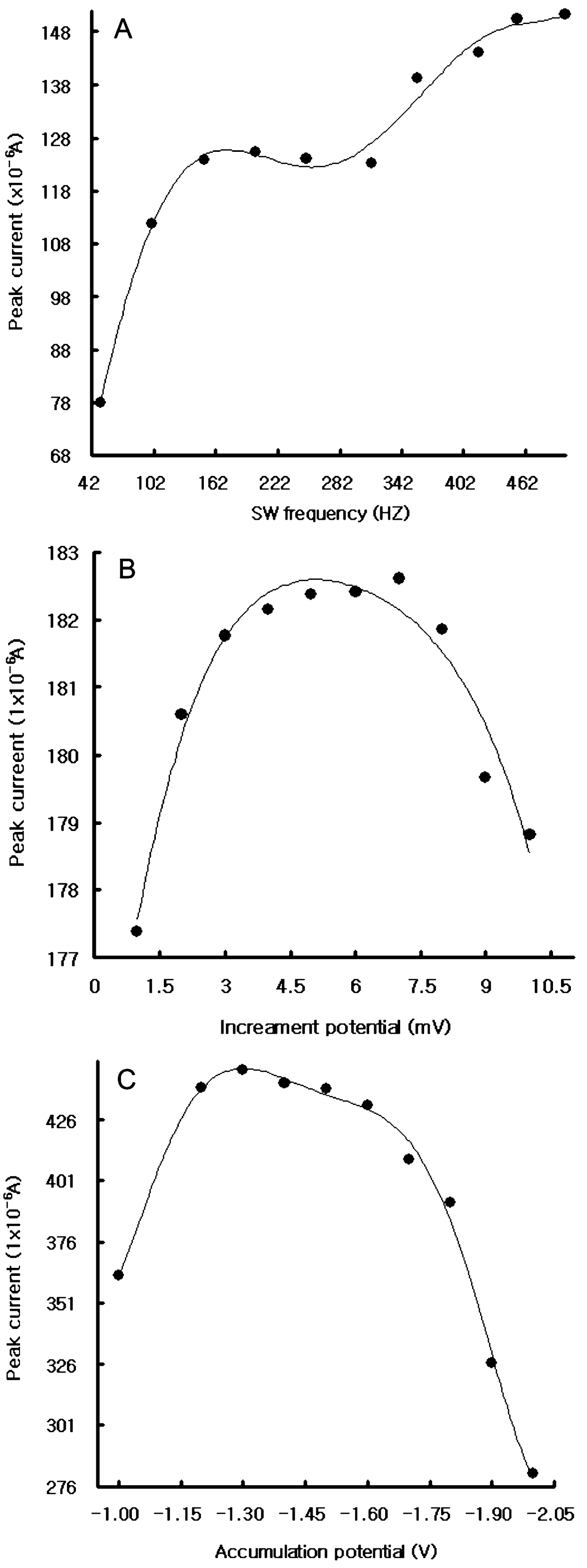

**Fig. 2.** (a) Peak currents of varying square wave frequencies of 50, 100, 150, 200, 250, 300, 350, 400, 450, and 500 Hz. (b) Incremental potentials of 1, 2, 3, 4, 5, 6, 7, 8, 9, and 10 mV. (c) Accumulated potentials of -1, -1.2, -1.3, -1.4, - 1.5, and -1.6.

and the 0.2 V peak potential very quickly increased from 50 to 150 Hz, and then to 312 Hz after the peak current did not increase. From 357 to 500 Hz, the 0.2 V peak current very quickly increased. Thus, all the other frequency conditions were used at 500 Hz; and at the maximum conditions, the 0.2 V peak current appeared at a 151.2 × $10^{-6}$ A high, whereas the 0.4 V peaks were smaller and later disappeared. Thus, all the other experimental conditions used these results. Fig. 2(b) shows the various incremental potentials of 1~10 mV when other parameters were used. In this voltammogram, two separate peaks appeared and the peak width broadly increased. Finally, the maximum response was obtained at the 7 mV incremental potential, at which a 181.86 × $10^{-6}$ A peak high appeared more sensitively than did the frequency results. Thus, this potential was used for all the other experimental conditions. In Fig. 2(c), the accumulated potentials were examined for the native potential range of -1~-2 V using the conditions in Fig. 2(b). At -1.3 V, maximum peak currents of 446.7 × $10^{-6}$ A were obtained, which responded better than the frequency or incremental potential peak high, and -1.3 V was used for all the other experiments, the peak sharps of which were not separated. Also, cathodic stripping was performed at the optimized conditions, but no signals were obtained.

Fig. 3(a) shows the results of various square wave amplitudes that were examined for the 0.01-0.35 V range of the peak potentials that appeared at 0.2 V, for other small peak potentials that responded positively at 0.5 V, and for whole peak currents that increased to 0.35 V, then did not increase. The peak width also broadly appeared. Thus, all the other conditions were used at the 0.35 amplitude potentials. At this state, an 80.23 × $10^{-6}$ A peak high was obtained, which was more poorly influenced than were the other stripping parameters. Finally, the accumulation times were examined. Fig. 3(b) shows the results, which responded very sensitively and better than the other experimental parameters. Thus, lower concentrations of 0.01 μg $L^{-1}$ Hg(II) were examined from 20 to 400 s accumulation times at the range of 0~20 sec. The oxidation peaks slowly appeared and the peak width broadly increased, whereas at the 50~400 s range, the peak high quickly increased and the peak half-width sharply decreased. At these conditions, the maximum current of 78.57 × $10^{-6}$ A appeared, no separate peak appeared, and only a simple peak was obtained. Increasing accumulation times were not used for the experimental time consumption at the fixed conditions, however, and the electrode-usable times were examined for several weeks, during which time the electrode surface was cleaned with the weighing paper and a much longer reproducibility was obtained. Finally, the analytical application was examined in the biological and toxicological waste samples.

***Statistics and application.*** At the optimized conditions, various working concentrations ranges were examined. Fig. 4(a) shows the results of a 400 s deposition time at these conditions. One to 12 ng $L^{-1}$ Hg(II) appeared linearly, and the peak width was very sharply obtained, whereas at the more increased concentrations of 13~15 ng $L^{-1}$, the mercury ion responded nonlinearly. All the raw and calibrated equations show this.

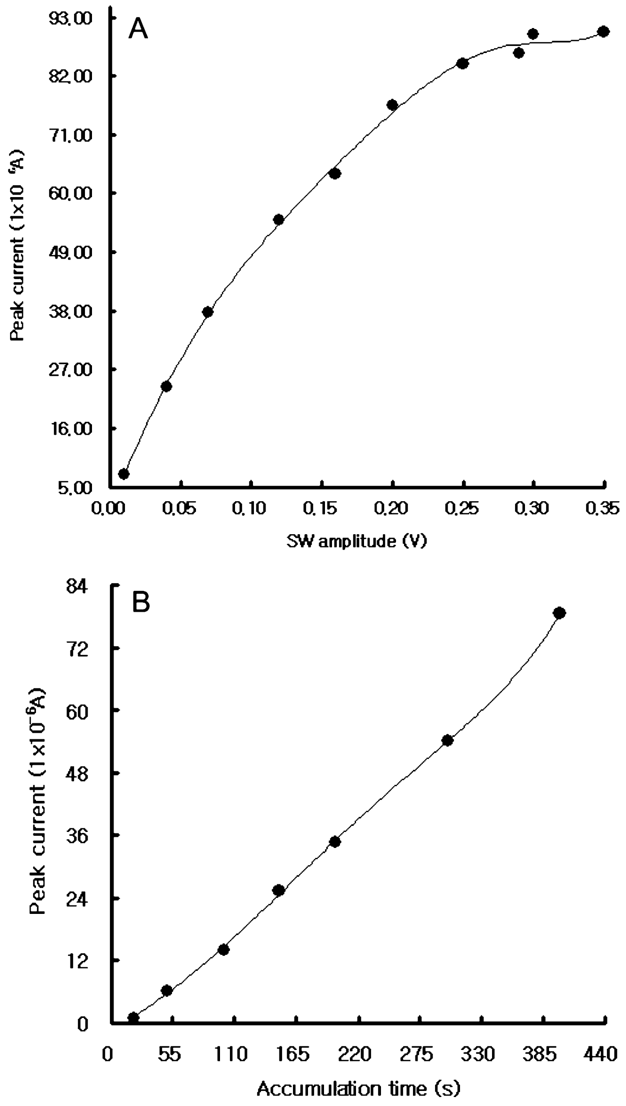

**Fig. 3.** (a) Square wave amplitudes of 0.01, 0.04, 0.07, 0.12, 0.16, 0.2, 0.25, 0.29, and 0.35 V in 100 mg $L^{-1}$ Hg(II). (b) Peak currents of varying square wave accumulation times of 20, 50, 100, 150, 200, 300, and 400 s in 0.01 $\mu$g $L^{-1}$ Hg(II). The other parameters in Fig. 4 were held constant.

At these ranges, a sensitive slope of $\Delta x/\Delta y = 1.4085$, a precision level of $R^2 = 0.9899$, a regression equation of y = current A, and an ng $L^{-1}$ concentration level of x = Hg(II) were obtained. Moreover, increased concentrations of microgram ranges were obtained (not shown here), and for the linear working ranges of 20, 30, 40, 50, 60, 70, 80, 90, and 100 ug $L^{-1}$, the equation $y = 0.3598x - 2.5837$ Hg(II) and the correlation coefficient of 0.9994 were obtained. At the optimum conditions, the analytical detection limits (S/N) were examined, and the

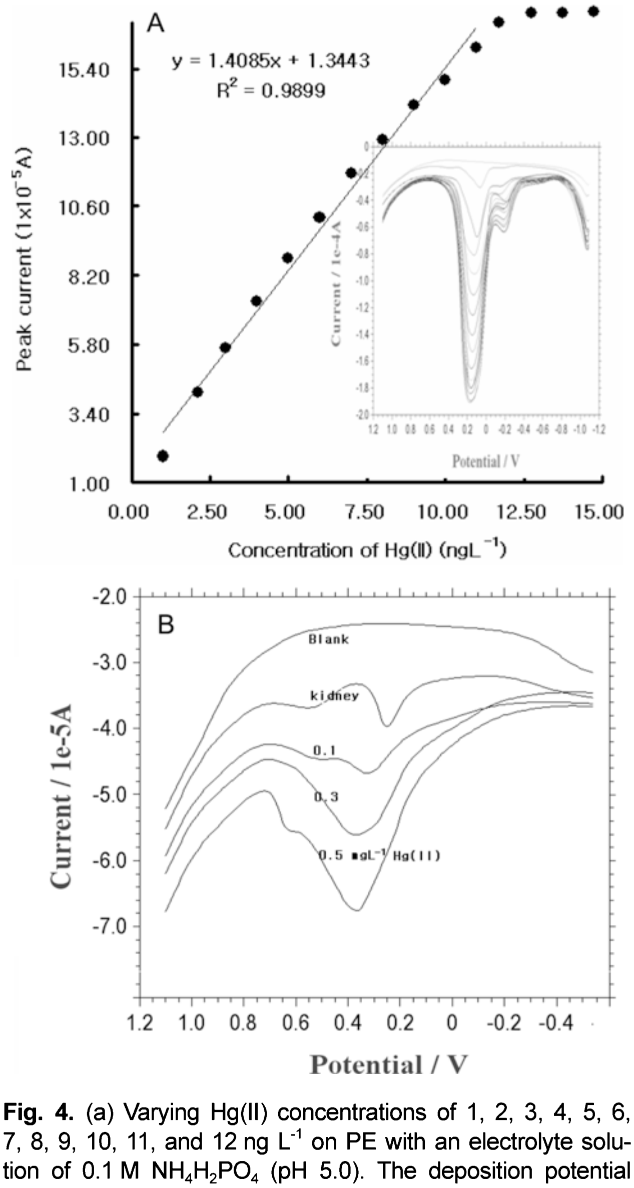

**Fig. 4.** (a) Varying Hg(II) concentrations of 1, 2, 3, 4, 5, 6, 7, 8, 9, 10, 11, and 12 ng $L^{-1}$ on PE with an electrolyte solution of 0.1 M $NH_4H_2PO_4$ (pH 5.0). The deposition potential was -1.3 V for a 400 s accumulation time, an SW frequency of 500 Hz, a 0.35 V amplitude, and a 7 mV incremental potential. (b) Application of the blank solution, the 0.03 m $L^{-1}$ fish kidney, and the 0.1, 0.2, and 0.3 mg $L^{-1}$ standard Hg(II) spikes. The other experimental parameters in Fig. 3 were held constant.

results yielded 0.2 ng $L^{-1}$ for Hg(II) ($7.37 \times 10^{-13}$ M). The experimental results were more sensitive than those derived from other common voltammetric methods, and from such results, various possible interference metal ions were examined by adding several other ions using 0.1 mg $L^{-1}$ Hg(II), and the existence of 1 mg $L^{-1}$ of Pb(II), Ba(II), Ca(II), Bi(II), Co(III), Fe(II), Cr(III), and Pt(I) resulted in 29.3%, 330.0%, 106.2%, 66.6%, -78.8%, 97.2%, -23.6%, and -61.0%. The presence of other ions was also effectively removed using standard addition methods. At the optimum conditions, the analytical applications were performed with river fish kidney solutions prepared using fish with 80 g body weights and extracted kidneys with 0.53 g weights, which were diluted in a 0.1 M HCl solution and examined during the application. In Fig. 4(b), however, the first peak shows the electrolyte solution results, after which the 0.03 ml sample solutions were spiked and a small 0.2 V mercury ion potential appeared, which manifested no noise signal. Thus, another standard for 0.1, 0.3, and 0.4 mg $L^{-1}$ Hg(II) was spiked at these results, calculated for the standard addition methods, and yielded $y = 0.0345x + 4.783$ and $R^2 = 0.9818$ and 0.139 mg/g. Other known waste solutions were examined and a mean 95% confidence limit was obtained via triple analysis. Quantitation of known laboratory waste samples was prepared with 1.0 μg $L^{-1}$ Hg(II) using an industrial solution. In a 10 ml 0.1 M $NH_4H_2$ $PO_4$ electrolyte solution, a spiked 1.5 ml waste sample showed a distinct peak current of Hg(II), after which 6, 12, and 18 μg $L^{-1}$ Hg(II) standards were added to the electrolyte cell systems. Three repeated determinations were made on each cell system. The plot of the peak current against the mercury concentration was linear ($R^2 = 0.998$). The concentration of mercury in the sample was found to have been $0.96 \pm 0.06$ μg $L^{-1}$ (n = 3). This agrees well with the value from the known concentration.

## CONCLUSION

A newly prepared DNA and carbon nanotube paste electrode was developed to detect mercury ions at nanogram levels with stripping and cyclic voltammetry. The method offers attractive properties compared to other voltammetric methods, such as a lower detection limit, simple electrode preparation, and long stability. Optimized analytical conditions were researched on and applied to the detection of mercury ion concentrations in low concentration ranges, and various interference ions were searched for. The analytical applications of the fish tissue were also examined. This study can also be applied in other fields that require mercury ion analysis of food or environmental toxicology.
